# Perspectives of Infant Mortality from African American Community Members

**DOI:** 10.1089/whr.2023.0017

**Published:** 2023-08-23

**Authors:** Kalyn M. Renbarger, Sheila Abebe, Jean Marie Place, Elizabeth Goldsby, Gabe Hall, Adam Kroot

**Affiliations:** ^1^ Department of Nursing and Ball State University, Muncie, Indiana, USA.; ^2^ Department of Public Health, Ball State University, Muncie, Indiana, USA.; ^3^ School of Nursing, Ball State University, Muncie, Indiana, USA.

**Keywords:** infant mortality, qualitative, African American

## Abstract

**Introduction:**

Infant mortality (IM) is often used to determine overall population health and well-being. Health disparities exist with African American (AA) infants having higher rates of IM than White infants. The purpose of this study was to examine the knowledge, attitudes, and perceptions of members in an AA community regarding IM, which can be used to develop interventions.

**Methods:**

A qualitative descriptive design guided this study. A county in the state of Indiana was the setting from which the researchers enrolled participants in this study. The participants consisted of 16 AA community members who were recruited from a local agency and who had completed an educational program on IM. Through semistructured phone interviews, participants described their understanding of IM. The data analysis of the transcribed interviews was performed *via* content analysis to yield overall themes from the data.

**Results:**

The analysis identified three themes describing AA Community members' perspectives on IM: (1) *Shying Away from the Topic of Infant Mortality;* (2) *Receiving Misinformation from Family Members;* and (3) *Considering Infant Mortality as Unpreventable.*

**Discussion:**

The findings of this study suggest that participants avoided the topic of IM, often received misinformation from family members, and believed infant death could not be prevented. Health care providers should have an open and culturally competent discussion about issues of IM, engage family members, and support community-based initiatives and education for members in AA communities.

## Background

The death of an infant has devastating consequences for the family, community, and society. Infant mortality (IM) is the term used to describe the death of an infant in the first year of life.^1^ The infant mortality rate (IMR) is the number of infants who die in the first year of life per 1000 live births.^1^ IM is recognized as a leading health indicator in the United States, indicating that IM reflects the overall nation's health and well-being.^2,3^

Overall, the IMR has trended downward since 1995 in the United States and decreased by 19% since 2005.^4^ More recently, the difference in IMR declined by 3% in 2019 to a rate of 5.58 compared with 5.67 in 2018.^4^ The leading causes of IM in 2019 included congenital malformations, disorders related to short gestation and low birth weight, maternal complications, sudden infant death syndrome, and unintentional injuries.^4^

While the overall IMRs are declining, a closer examination of data revealed devastating health disparities, with African American (AA) infants at the highest risk. The IMR for Black infants was 10.62 compared with that of Hispanic (5.03) and White infants (4.49) in 2019.^4^ Indiana is an area of particular concern for IM. Indiana's overall IMR was higher at 6.5 versus the nation's IMR at 5.6 in 2019.^5^ The trend in disparities in Indiana reflects what is occurring nationally, with Black infants twice as likely to die in the United States than White and Hispanic infants.^5^ Black infants in Indiana are more likely to be born preterm or with low birth weight, placing them at greater risk for IM.^5^

Social determinants of health, conditions in the places in which individuals live that affect health risks and quality-of-life outcomes, have been linked to IM in Black infants.^6^ Social inequities such as access to health care, socioeconomic disadvantage, and contextual disparities within education levels may be contributors to poorer health outcomes in Black infants compared with White infants.^2^

Racism, discrimination, and mistrust in health care providers have hindered Black women from seeking prenatal care.^7^ In addition, it has been reported that Black women are 2.1 times more likely than White women to receive prenatal care late in their pregnancy or not at all.^8^ Inadequate prenatal care has been associated with increased odds of stillbirth, preterm birth, low birth weight, and small for gestational age.^9^

The social determinants of health influence attitudes, knowledge, and behaviors related to IM prevention measures such as breastfeeding and infant safe sleep practices. Breastfeeding is considered the gold standard of infant nutrition.^10^ It is essential to understand how social determinants of health impact breastfeeding practices and influence IM in AA infants. Breastfeeding initiation has been associated with decreased risk of infant death in diverse racial and ethnic groups in the US population.^11^

However, Black women have lower rates of breastfeeding than in other populations.^12^ Black women have reported receiving little assistance with breastfeeding from health providers.^13^ The interplay of family members, myths, and the internet “as my friend” was more influential. The extended family and kin networks play an important role in childcare. It has been reported that AA women often look to their mother and maternal grandmother for parenting information and reinforcement, which may serve to support or discourage best practices.

Grandmothers who are misinformed about current childcare recommendations may diminish a mother's confidence in caring for her infant.^14^ Tran et al.^15^ discovered barriers to breastfeeding in Black women, which included inaccessible lactation support and supplies, perceptions of breastfeeding as time-consuming, lack of access to and knowledge of breastfeeding laws and policies, and negative cultural norms or stigma.

Sudden unexplained infant death (SUID) is another contributing factor to IM, which can result from an unsafe sleep environment with risk factors, including sleep position, bed sharing, soft bedding, sleep surfaces, and maternal smoking.^16^ Moreover, in a study examining infant sleep practices between US Hispanic and AA families, results showed that despite similar socioeconomic statuses, AA infants are at a higher risk of sudden death,^17^ suggesting that cultural differences are contributing factors at play.

Research by Tully et al.^18^ suggested that despite many AA women reporting an understanding of safe sleep practices, several factors negatively influence their infant sleep practices. For instance, many AA women reported perceived safety, convenience, quality of infant sleep, unplanned bed sharing to soothe nighttime fussiness, and conflicting information from family members as reasons for noncompliance.^18,19^

Knowledge, attitudes, and perceptions regarding IM can influence health behaviors and outcomes and thus are important factors in determining appropriate interventions. In efforts to implement the most effective interventions, it is necessary to understand the knowledge, attitudes, and perceptions of AA community members about IM. Having an understanding of AA community members' knowledge, attitudes, and perceptions about IM is crucial for health care providers to address all behaviors that could negatively impact IM.

To date, there is a lack of studies investigating the attitudes, knowledge, and perceptions of the AA community regarding IM. IM in this study was viewed through the lens of a life course perspective. A life course perspective, which begins with exposure *in utero*, was used to identify conditions that influence the development of health disparities related to AA IM. A life course perspective acknowledges that health status reflects cumulative life conditions.^20^

## Methods

### Aim

The purpose of this study was to examine the knowledge, attitudes, and perceptions of members in an AA community regarding IM.

### Design

A qualitative descriptive approach was used to produce findings that focus on the surface meaning of participants' words to describe a phenomenon.^21^ The approach provides data in a direct format that can be used to inform health care, policy, and practice. Sandelowski^21^ offered examples of questions using a qualitative approach, which addressed the concerns of people about an event, peoples' responses (*e.g.*, thoughts, feelings, and attitudes) toward an event, and the factors that facilitate or hinder recovery from an event.

Qualitative description was determined to be the most appropriate method because the goal of this study was to provide straightforward descriptions of IM from AA community members who completed an online educational program. The educational program consisted of four online modules on the topics of breastfeeding, smoking cessation, infant safe sleep, and accident prevention.

The breastfeeding module consisted of a 20-minute video that covered the benefits of breastfeeding, common breastfeeding myths, barriers to breastfeeding in AA women, and resources to support breastfeeding women in the community.

At the completion of the program, participants were invited to participate in an in-depth interview to assess attitudes, knowledge, and perceptions regarding IM.

### Sample

The sample included 16 participants who were recruited from a county in Indiana. The inclusion criteria for this study were patients who (1) were aged 18 years or older; (2) identified as AA; (3) could speak and write in English; (4) resided in a particular county in Indiana; (5) completed an online educational program on IM, and (6) consented to be audio recorded. To obtain an understanding of IM in the AA community, participants were randomly selected if they met the inclusion criteria.

Participants could identify as any gender and both parenting and nonparenting individuals were included in the sample. The county in Indiana was selected for data collection based on the location of the community education intervention. The selected county has IM rates that are above the average for both Indiana and the United States.

### Procedures

This study was reviewed before beginning by the Institutional Review Board of the first author's institution and qualified for an exemption. A study announcement that briefly described the study was widely distributed to local agencies and posted on social media sites. Interested participants were invited to call a local partner agency to participate in the online educational modules. Upon completion of the educational program, participants were contacted by the research team to set up a time for an in-depth interview.

The research team consisted of three doctoral-prepared nurse researchers, a doctoral-prepared community health specialist, and two graduate students, all of whom were experienced in maternal–child health and qualitative research. Participants provided verbal consent. The researchers conducted audio-recorded phone interviews using an interview guide (see Appendix A1), and each interview lasted between 30 and 40 minutes.

The interviews were conducted from December 2021 to April 2022. Participants were asked to describe their knowledge and perceptions regarding IM in the AA community. Participants were given a $100 gift card after completing the educational program and interview. To ensure confidentiality, audio recordings were stored in a password-protected computer, and participant information was deidentified and labeled with a study identification number.

### Analysis

Data analysis was completed by the first and second authors using a content analysis approach, supported by NVivo.^22^ A content analysis was conducted by Kyngäs^23^ because the data collection approach was open and followed loosely described themes. Kyngäs^23^ described the following steps to a basic inductive content analysis: data reduction, data grouping, and formation of concepts that can answer the research question. The researchers read through all transcripts to become immersed in the data.

In the first step of the data analysis, the first author highlighted and extracted each text unit (*e.g.*, phrase, sentence, or story) related to the participants' accounts of IM. Each text unit was assigned a code by the first author. Next, during the data abstraction process, open codes were analyzed for similarities and differences by the research team and then grouped to form themes and subthemes. Last, descriptions of IM in the AA community, which included exemplar quotes taken from the transcripts, were developed by the first and third authors.

The descriptions of IM were reviewed by the research team, and consensus was reached. An audit trail that chronicled all methodological decisions was maintained by the first author. The research team discussed their potential biases and the context in which the study was conducted throughout the data analysis process. The data were considered saturated after 16 participant interviews as no additional themes emerged.

## Results

All participants identified as AA, were 18 years of age or older, and lived within a county located in Indiana. Eleven participants identified as female and five identified as male.

### Participants' descriptions of IM

Three themes emerged from the data to demonstrate participants' perspectives on IM: *Shying Away from the Topic of Infant Mortality, Receiving Misinformation from Family Members, and Considering Infant Mortality as Unpreventable.*

#### Shying Away from the Topic of Infant Mortality

In the first theme, several participants expressed the sentiment that IM is “out of sight, out of mind.” Participants spoke of minimizing the issue of IM or ignoring the subject altogether. One participant (p1) stated, “We've never had IM, so therefore, it's not as big of a deal as people make it out to be or whatever.” One participant (p1) said, “We tend to ignore it [infant death], or if it's not a part of my world, it doesn't exist.”

Another reason for shying away from the topic of IM included participants' desire to avoid things that are negative or traumatic. Participants did not desire to think about an infant dying. One participant (p3) believed that avoiding discussions on IM contributed to the problem and indicated,

“We don't want to hear about that [infant death]. We don't want to talk about that or watch videos. They automatically thought the worst, but the absolute worst part is it's [infant death] because we aren't educating ourselves. People don't want to believe that bad things are happening for whatever reason.”

Another participant (p4) suggested that most individuals anticipate a healthy infant and avoid speaking about IM and stated,

“I think it's [infant death] an unspoken concern. We like to think that things are going to be perfect, things like that, and you don't want to talk about bad realities. It's [concerns about IM] unspoken until it [infant death] happens.”

While participants avoided speaking about IM, they believed they would be faced with speaking about IM if they or someone close to them had a personal experience with infant death. Another participant suggested that in the absence of personal experience, it is important to talk about IM on billboards or other public spaces so that individuals are forced to acknowledge that IM is a serious issue in the AA community.

#### Receiving Misinformation from Family Members

In the second theme, participants were sometimes hesitant to seek advice from health care providers and thus relied on family members. Many participants discussed the dual role that grandmothers and other extended family members played. Grandmothers had helpful tips, tricks, and techniques that helped infants sleep longer, such as swaddling.

However, other recommended techniques were unsafe, such as putting infants to sleep on their stomachs. One participant (p5) noted, “You can sometimes probably have a grandmother who's older that'll say, ‘Oh, the baby will be fine sleeping on their stomach. We done it for years and nothing happened to you.’” Other participants reported having to navigate conflicting recommendations. One participant (p6) stated,

”I just feel like the older generation thinks because this worked back in the day that it's still appropriate, but studies are showing differently. They give advice, but it's taken with a grain of salt.”

Some participants did not feel comfortable accepting advice from older family members. One participant (p7) sought other sources and stated, “The older generation has outdated ideas and that it is probably better to get expert opinions or looking things up on the internet,” while another participant (p8) acknowledged, “A lot of times we learned from our parents how to care for an infant and a lot of those things are no longer supported by research to be safe.”

As one participant (p9) noted, “not all help [from extended family members] is good help,” participants had varying ideas of where to access correct information. Answers ranged from seeking out health care professionals, consulting Facebook and other internet sources, or talking to other mothers.

#### Considering Infant Mortality as Unpreventable

In the third theme, participants were unsure as to whether IM could be prevented. Participants largely attributed infant death to illness and it was seen as something they could do little in advance to prevent. One participant (p10) stated, “If the baby already had a certain illness already, to begin with, yes. I don't think it [the illness] helps the situation.”

A fatalistic mindset limited participants from acknowledging factors that affect IM, such as behavioral and environmental risk factors in the pre- and postnatal periods (*i.e.*, smoking, diet, stress, and safe sleep practices). One participant stated (p11), “Some of that stuff, you know, can't be prevented when it comes to IM,” while another participant (p3) expressed, “It's just seeing babies not growing, babies dying way, way before their time, but there's no real like pinpoint explanation for it [infant death].”

Some participants correctly noted SUID as a reason for IM; however, no participants indicated that there are preventive measures to reduce the risk of SUID (*i.e.*, putting the baby in a safe sleep environment). One participant (p12) stated, “I mean as far as just stop breathing, you can't help something like that.” Another participant (p13) indicated, “See, as I know, it's just the sudden death thing where you can't do anything about that, or can you?”

## Discussion

The findings of this study underscore the cultural influence of family members in the AA community, which resonates with prior literature. Stiffler et al.^24^ indicated that family members often encouraged AA mothers to have their infants sleep with them. The authors also noted that some participants were a part of African cultures that had family beds, so they expected the infants to sleep with the family.

In another study, AA participants identified generationally persistent traditions related to infant feeding. One participant discussed the impact of generational traditions on her infant-feeding choice, stating “Everyone in my family bottle feeds, it's just been generationally. That is the way Grandma did it and it continued.”^25^

The findings also revealed that participants tended to shy away from discussing IM due to viewing IM as not preventable. This echoes another study where AA participants described experiences of themselves or others where they did everything right and still had adverse pregnancy outcomes.^26^

Additionally, participants minimized the risk of infant death. In a systematic narrative synthesis, Ward^27^ discovered that parents exhibited disagreement and skepticism about the dangers of bed sharing. Potential risks of infant death were minimized by parents because they felt that the risks did not apply to them because they considered themselves “light” sleepers and would “never” roll onto their infant.

Other studies have also found that there continues to be a gap in knowledge of SUID and the practice of protective behaviors in AA women. For example, AA women have not found a possible relationship between sleep practices and SUID. The AA women considered SUID to be “God's will” and that changing their behavior would not prevent the death from occurring.^17,28^

### Limitations

The findings of this study should be considered in the context of limitations. First, the sample size was small and from one geographical location. The small sample size did not allow for more distinct interpretations such as the influence of age, gender, and family role on participants' descriptions of IM. Second, it is also not known if the results can be broadly applied to other AA populations. Although the sample size was small, data saturation was achieved.

Future research should include a more robust sample size to better understand AA community members' perspectives on IM. We acknowledge that the sample of AA participants in our study may differ from other AA populations elsewhere in the United States. Nevertheless, because of the sparsity of data on knowledge, attitudes, and perceptions among this population, this study adds to the literature base.

### Implications for providers

The findings of this study have several implications for health care providers to promote cultural competency. First, it is important for health care providers to understand the role that culture plays in AA families compared with other populations. Health care providers must understand the role of grandmothers and extended family. Moreover, health care providers must be flexible, respectful, and supportive about an individual's culture, creating space to reframe, without judgment or paternalism.^14^

Second, education is needed on issues related to IM and it should incorporate culturally sensitive messaging. Health care providers should have open discussions and remain nonjudgmental regarding issues of IM, such as where and how the infant sleeps and infant feeding decisions, by (1) engaging family members (including partner) in discussions; (2) inviting the family to share the most relevant influences regarding their decisions for infant care; and (3) providing additional information to support their decisions, including precautions to take if their decisions in infant care are not supported by research, such as bed sharing, in preventing IM.^29^

Third, health care providers can support initiatives for more community involvement to address issues of IM in AA communities. Participants were reluctant to seek advice from health care providers and may be more prone to discussions with other AA community members. Gaydos et al.^19^ discovered that health care providers expressed an inability to effectively counsel AA women even when the providers themselves were AA, were in locations with largely minority populations, and trained in cultural competence.

Finally, considering that AA women are hesitant to seek advice from health care providers, community-based education is critical. Community-based education should be clear and include illustrations on the topic, reinforced by various teaching platforms (*i.e.*, social media and formal education), and conducted in small groups.^30^ Furthermore, messaging on IM must acknowledge AA mothers and consider their beliefs and understanding behind their practices on IM to be effective and encourage change.^31^

## Conclusions

This study revealed three themes to describe AA community members' perspectives on IM. Participants minimized the topic of IM, believed that IM could not be prevented, and described receiving misinformation from family members. Recommendations for health care providers include having open, honest, and culturally competent discussions about issues related to IM; engaging family members in such discussions; and supporting community-based initiatives to address IM in AA communities.

## Abbreviations Used

AA: African American
IM: infant mortality
IMR: infant mortality rate
SUID: sudden unexplained infant death

## Appendix

### Appendix 1

*I want to thank you for participating in this study. I am interested in your understanding and experiences related to infant mortality. You may choose to not answer any questions that you do not feel comfortable answering. Before we begin, do you consent to participate in this study? Do you consent to have the interview/focus group audio recorded?*

1. What comes to mind when I say the term, “infant mortality?”
2. What are the things that women/mothers need to have a healthy pregnancy and healthy baby?
3. What are the behaviors or practices of parents/caregivers that can help keep a baby safe?
4. I have listed some items. Let me know if you think the items contribute to keeping a baby safe or not:
  i. Tobacco/smoking/other substance use
  ii. Sleeping practices (*e.g.*, sleeping in the same bed, sleeping in the same room, using bumper pads and other things in cribs, babies going to sleep with a pacifier, babies going to sleep on their back, firm mattress, no loose bedding, sleep sacks).
  iii. Breastfeeding
  iv. Relationship stress/violence
  v. Bottle propping
  vi. Accidents (car seat safety)
5. What do other members of the family (grandparents, aunts, *etc*.) think about caring for a baby? Do they have advice or dos/don'ts that they talk about?
6. What are the challenges or difficulties you think African American parents in this county, in particular, might experience in taking care of their baby and keeping the baby healthy?
7. Are there things about this county that make it easier for moms and dads to take care of their baby?
8. Infant mortality (or the number of babies who die before the age of one) is higher in the African American community than in White communities. Why do you think that is?
9. Where do parents or caregivers get trusted information on how to raise a healthy baby?
10. Do you think infant mortality is preventable? Why or why not?
  a. Do you think other people think it is preventable? Why or why not?
11. What are the questions that moms, dads, or caregivers in the African American community might have about taking care of their child during pregnancy or after, which they might not feel comfortable asking?
12. How concerned are people you know about infant mortality?
13. Is there anything else that you think is important for other parents or other caregivers to consider to prevent infant mortality?
